# A 266-nm multi-reflection laser curtain for airborne bacterial inactivation in a 20-m^3^ chamber and a single-pass duct testbed

**DOI:** 10.3389/fpubh.2026.1809909

**Published:** 2026-08-25

**Authors:** Chenjie Guo

**Affiliations:** Department of Medical Equipment Management, Shanghai Pudong New Area Hospital of Traditional Chinese Medicine, Pudong New Area, Shanghai, China

**Keywords:** air disinfection, bioaerosol, in-duct disinfection, infection prevention and control, ultraviolet germicidal irradiation

## Abstract

**Background:**

Airborne transmission remains a challenge for infection prevention and control in healthcare environments, motivating compact air-disinfection technologies.

**Methods:**

A compact 266 nm multi-reflection laser-curtain module was developed, providing two effective curtain widths (96 mm and 200 mm). Aerosolised Staphylococcus albus (S. albus; a coagulase-negative staphylococcal bacterial surrogate) was tested in (i) a mixed-air 20 m^3^ enclosure for time-dependent inactivation and (ii) a duct-flow single-pass rig using three curtains in series with paired upstream-downstream sampling. Matched no-UV controls were used for baseline correction, with first-order kinetics used for room-scale analysis.

**Results:**

In the 20 m^3^ enclosure, inactivation increased with operating time. The 96 mm curtain achieved ~66–69% at 5 min and exceeded 98% by 30 min, approaching ≥99.9% with extended operation. The 200 mm curtain exceeded 99.5% by 20 min and approached ≥99.99% at longer times, with a numerically higher apparent rate constant (0.190 vs. 0.127 min^−1^). In the duct-flow system, three curtains maintained ≥99.0% single-pass inactivation across 1.2–9.6 m/s.

**Conclusions:**

The multi-reflection 266 nm laser-curtain achieved high inactivation in controlled room-scale and duct single-pass tests, supporting further engineering evaluation of this air-disinfection concept rather than immediate healthcare deployment.

## Highlights

- Developed a compact 266 nm multi-reflection laser-curtain module that provides a distributed UV treatment region across the airflow cross-section.
- Demonstrated time-dependent inactivation of aerosolised Staphylococcus albus in a mixed-air 20 m^3^ chamber, with a numerically higher apparent rate constant observed for the wider curtain configuration.
- Achieved ≥99.0% single-pass inactivation in a duct-flow testbed using three curtains in series across air speeds of 1.2–9.6 m/s.

## Introduction

Airborne transmission is increasingly recognized as a persistent route for the spread of respiratory and other pathogens in healthcare environments, particularly where close contact, high patient turnover and aerosol-generating activities coexist (1). Ventilation remains a foundational engineering control, but its realized effectiveness depends on ward geometry, occupancy, air distribution patterns and operational constraints that are often difficult to optimize in multi-bed clinical spaces (1). These practical limitations motivate adjunct technologies that directly inactivate bioaerosols in air, complementing rather than replacing ventilation-based mitigation.

Ultraviolet germicidal irradiation (UVGI) has a long history as an air disinfection approach supported by a well-established biophysical rationale and extensive applied experience (2). In healthcare facilities, UVGI is generally positioned as an effective adjunct when integrated appropriately into facility design and infection prevention workflows, while it is not regarded as a stand-alone solution (3). Upper-room UVGI has been evaluated in real-world contexts aimed at reducing transmission of airborne infections, demonstrating that clinically relevant deployment is feasible when systems are suitably designed and operated (4). Renewed attention to airborne spread in the context of SARS-CoV-2 has further reinforced the importance of layered mitigation strategies and the potential role of air disinfection in higher-risk indoor environments (5, 6).

Despite this foundation, translating UV-based air disinfection into robust, deployment-oriented solutions in hospitals remains non-trivial. Contemporary assessments of ultraviolet-C technologies in clinical environments emphasize that effectiveness and safety are contingent on engineering details, installation conditions, and maintenance practices that can vary substantially between sites (7). For room-scale implementations, performance is additionally shaped by airflow mixing and the frequency with which aerosol parcels traverse the irradiated region, making cross-study comparison challenging unless test conditions and reporting metrics are standardized (8, 9). These considerations highlight the need for device concepts that provide a well-defined treatment region and for evaluation approaches that enable consistent interpretation across room-scale and ventilation-relevant settings.

In-duct UVGI provides an implementation pathway that leverages ventilation ducts to deliver controlled exposure during airflow transit. Design analyses and reviews of in-duct UVGI underscore that delivered dose depends on duct geometry, air velocity, residence time, emitter arrangement, and the spatial uniformity of irradiance, which together govern single-pass performance and scaling to real ventilation systems (8). The strong dependence on residence time and dose distribution motivates architectures that can generate a spatially distributed irradiation field across the airflow cross-section and support more reproducible exposure characterization under controlled flow conditions (10).

Recent UVGI literature published after the COVID-19 pandemic has further emphasized that UV air-disinfection performance should be interpreted together with optical dose distribution, airflow behavior, aerosol transport, and system context. Reviews and design studies have examined rapid UVC air inactivation, HVAC duct configurations incorporating optically shaped UVC channels, and stand-alone UVC systems for indoor airborne pathogens (11–13). Applied HVAC studies and recent modeling work have also shown that relative humidity, lamp arrangement, air circulation devices, and coupled radiation-flow effects can substantially affect bioaerosol inactivation (14–16). These studies, together with broader reviews of emerging UV disinfection applications (17), support the need to link device physics, exposure conditions, airflow regimes, and microbiological endpoints when evaluating new air-disinfection technologies.

Against this background, there remains a need for compact UV air-disinfection architectures that can deliver a stable, distributed irradiation region across an airflow cross-section and can be assessed in both room-scale cumulative-treatment and ventilation-relevant single-pass regimes. The present study addresses this need by developing a 266-nm multi-reflection laser-curtain module and evaluating its airborne bacterial inactivation performance in two controlled experimental settings: a mixed-air 20 m^3^ chamber and a duct-flow single-pass testbed. The study focuses on process and device validation, with performance reported using room-scale kinetic parameters and duct single-pass inactivation metrics.

## Methods

### UV laser-curtain module

A compact deep-UV laser-curtain module operating at 266 nm was developed for airborne bacterial inactivation. The irradiation source was an in-house-built pulsed 266-nm ultraviolet laser with a pulse width of 10 ps, a repetition rate of 200 kHz, and adjustable output power. The measured optical power before the multi-reflection path was 0.77 W under the operating condition used for the attenuation measurement (Supplementary Table S2). At the laser-head facet, the beam diameter was approximately 5.16 mm and the beam divergence was approximately 18 mrad. Local irradiance verification was performed using an LS125 UV irradiance meter (Shenzhen Linshang Technology Co., Ltd., Shenzhen, China). A single measurement position was located near the first reflection point of the multi-reflection optical path, corresponding to the initial local irradiation region formed after the 266-nm beam entered the reflection chamber. The detector surface was oriented toward the local laser-curtain and reflected-beam region. This measurement was used as a local verification of the irradiation level and was not used as a spatially averaged irradiance measurement across the entire treated cross-section. No replicate LS125 measurements were performed; therefore, no mean, standard deviation, or measurement variability was available for the local irradiance reading. The nominal instrument precision was 0.01 mW/mm^2^. The instrument was within its valid metrological verification period from 6 November 2025 to 5 November 2026, with certificate No. 802811242. The wavelength of 266 nm is close to the nucleic-acid absorption maximum around 260 nm, providing the physical basis for UV-mediated microbial inactivation (2, 3).

**Figure 1:**
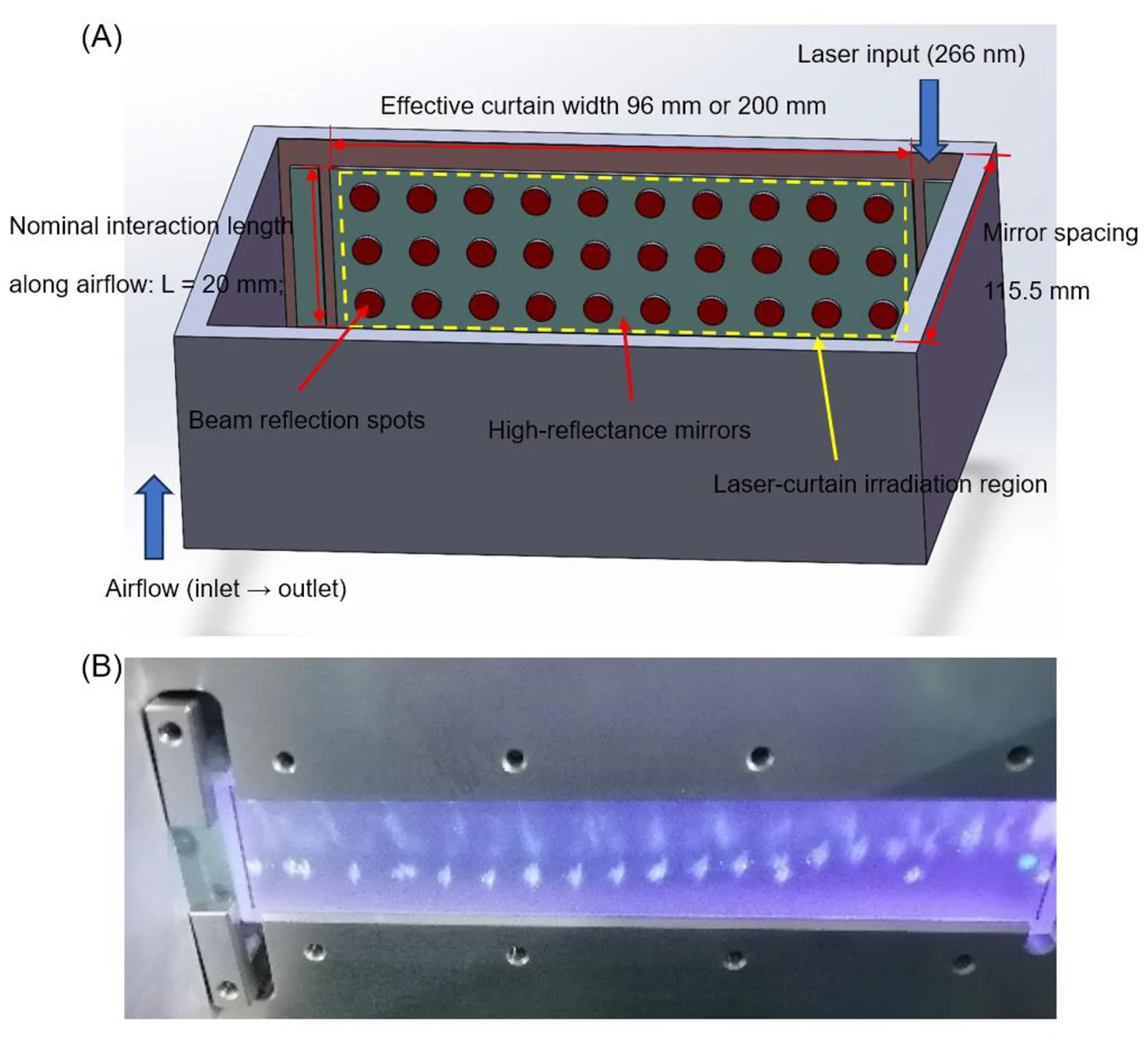
Laser-curtain module and irradiation geometry. **(A)** CAD rendering of the multi-reflection chamber showing the 266-nm laser input, airflow direction from inlet to outlet, UV-grade high-reflectance mirrors, representative beam-reflection points, and the laser-curtain irradiation region. The effective curtain width was 96 mm or 200 mm depending on the tested configuration, the nominal interaction length along the airflow direction was 20 mm, and the mirror spacing was 115.5 mm. The dashed outline indicates the laser-curtain irradiation region formed between the two high-reflectance mirrors. **(B)** Photograph of the prototype showing the visible UV laser curtain.

The optical core of the module was a multi-reflection chamber in which the incident beam was folded between two parallel UV-grade high-reflectance mirrors to generate a distributed irradiation region across the airflow cross-section (Figure 1). Two effective curtain widths, 96 mm and 200 mm, were implemented by configuring the illuminated width of the laser-curtain region. In the duct-flow configuration, the nominal interaction length of each curtain along the airflow direction was 20 mm, and the effective irradiation width used for dose estimation was 200 mm. For duct-flow single-pass experiments, three identical laser curtains were arranged in series to extend the cumulative interaction time during one transit through the test section, consistent with dose-residence-time considerations in in-duct UVGI evaluation (8, 10). A ray-tracing illustration of the folded optical path and laser-curtain formation is provided in Supplementary Figure S1. Optical power attenuation during repeated mirror reflections is reported in Supplementary Table S2; the average reflectivity estimated from these attenuation measurements was 98.84% per reflection.

### Test organism and overall experimental design

**Figure 2:**
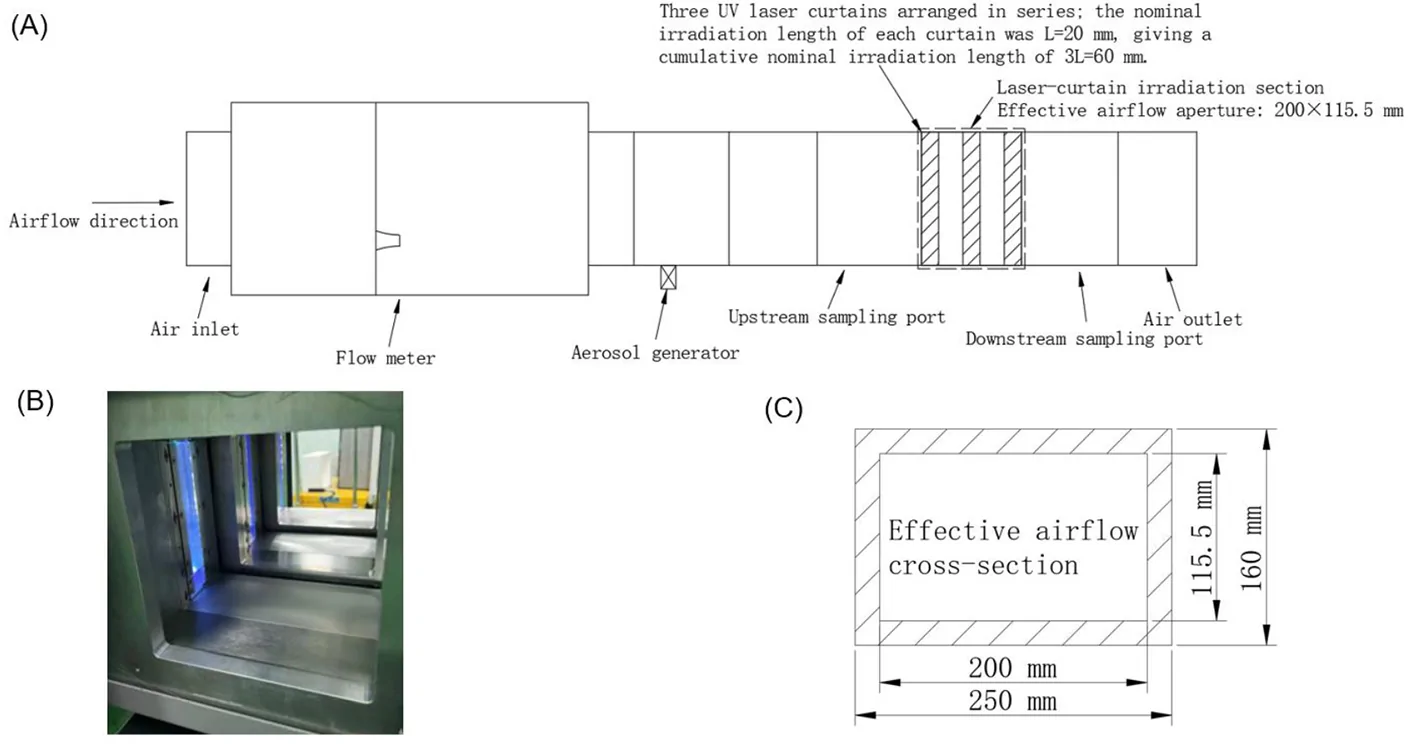
Duct-flow single-pass experimental set-up for aerosol inactivation. **(A)** Schematic of the test rig showing the airflow direction from the air inlet to the air outlet, flow meter, aerosol generator, three UV laser curtains arranged in series, and paired upstream and downstream sampling ports located immediately before and after the irradiation section. Each curtain had a nominal interaction length of 20 mm along the airflow direction, giving a cumulative nominal interaction length of 60 mm. The effective airflow aperture through the laser-curtain irradiation section was 200 mm × 115.5 mm. **(B)** Photograph of the laser-curtain modules installed in the duct section. **(C)** Cross-sectional geometry of the duct showing outer dimensions of 250 mm × 160 mm and an effective airflow aperture of 200 mm × 115.5 mm, corresponding to an effective airflow cross-sectional area of approximately 0.0231 m^2^.

All experiments were performed using aerosolised Staphylococcus albus (S. albus). The organism name is retained because it was the designation used for the experimental strain; taxonomically, S. albus is a historical designation commonly associated with coagulase-negative staphylococci such as Staphylococcus epidermidis. It was selected as a culturable bacterial surrogate for evaluating airborne UV susceptibility and device performance under controlled laboratory conditions. The use of a single bacterial species was not intended to represent viruses or the full range of clinically relevant pathogens. Two experimental scenarios were evaluated: (i) time-dependent inactivation in a 20 m^3^ enclosed chamber operated under mixed-air conditions, and (ii) single-pass inactivation in a duct-flow test rig with paired upstream and downstream sampling ports positioned immediately upstream and downstream of the irradiation section (Figure 2). For both scenarios, matched no-UV control trials were conducted under otherwise identical operating conditions to quantify baseline losses unrelated to UV irradiation and to enable correction of UV-attributed effects. Unless otherwise stated, each condition was repeated at least three times. The principal experimental parameters are summarized in Table 1.

**Table 1:** Summary of principal experimental parameters.

Parameter	Room-scale chamber experiment	Duct-flow single-pass experiment
Test organism	Aerosolised S. albus bacterial surrogate	Aerosolised S. albus bacterial surrogate
UV configuration	Single laser curtain; effective curtain width of 96 mm or 200 mm	Three laser curtains arranged in series
Test environment	20 m^3^ enclosed chamber; 20–25 °C; 50–70% relative humidity	Duct-flow testbed; air speed 1.2–9.6 m/s
Aerosol handling	Fan-assisted mixing for 5 min followed by 5 min stabilization	Aerosol introduced upstream with concurrent upstream/downstream sampling
Target concentration/ sampling	Approximately 5 × 104 to 5 × 105 CFU/m3; 28.3 L/min sampling flow	Baseline-corrected paired culture sampling; 28.3 L/min sampling flow
Replication and controls	At least three replicates per time point with matched no-UV controls	Three replicate single-pass measurements per airflow condition with no-UV transport-loss controls

### Room-scale experiment in a 20 m^3^ chamber

Room-scale experiments were conducted in a 20 m^3^ enclosed chamber at 20–25 °C and 50–70% relative humidity. Bacterial aerosols were introduced into the chamber, mixed by fan-assisted circulation for 5 min, and then allowed to stabilize for 5 min before sampling. This mixed-air chamber protocol was used to evaluate cumulative treatment under controlled room-scale conditions, recognizing that room-scale UVGI performance depends on airflow mixing and repeated traversal of the irradiated region (8, 9). The target initial airborne bacterial concentration was approximately 5 × 104 to 5 × 105 CFU/m^3^. Airborne bacterial concentration was calculated from colony counts using a sampling flow rate of 28.3 L/min.

For UV-treatment trials, the laser curtain was operated continuously, and samples were collected at predefined operating times. For the 96-mm-wide curtain, samples were collected at 5, 15, 30, 45, 60, and 75 min. For the 200-mm-wide curtain, samples were collected at 20, 30, 40, and 60 min. Matched control trials were performed using the same protocol with the laser curtain inactive. The two curtain configurations were evaluated using the same experimental workflow.

### Duct-flow single-pass experiment

Single-pass experiments were conducted in a dedicated duct-flow system comprising an inlet section, flow conditioning and measurement, an aerosol generation section, a laser-curtain treatment section, and paired upstream and downstream sampling ports (Figure 2). The treatment section contained three laser curtains arranged in series. For each airflow condition, the system was stabilized at the target flow setting before sampling. Control trials were first performed with the laser curtain inactive to quantify baseline transport loss across the test section. UV-treatment trials were then conducted under otherwise identical conditions with the laser curtain active. Upstream and downstream samples were acquired concurrently to minimize bias due to temporal concentration drift. Air speed was varied across representative duct-flow conditions from 1.2 to 9.6 m/s. The effective airflow aperture through the irradiation section was 200 mm × 115.5 mm, corresponding to a cross-sectional area of approximately 0.0231 m^2^. Exposure was interpreted in relation to the nominal residence time within the irradiation region, consistent with standard in-duct UVGI dose considerations (8, 10).

### Performance metrics and kinetic modeling

For room-scale experiments, UV-attributed inactivation was calculated by correcting the treatment-group concentration decay using matched no-UV control trials. Let *C*_*c*, 0_ and *C*_*c, t*_ denote the airborne bacterial concentrations in the control group at baseline and at time *t*, respectively. Let *C*_*UV*, 0_and *C*_*UV, t*_ denote the corresponding concentrations in the UV-treatment group. The natural decay fraction in the control group was defined as:


$$\begin{array}{l} N_{t}=\frac{C_{c,0}-C_{c,t}}{C_{c,0}} \end{array}$$

The UV-attributed inactivation efficiency was then calculated as:


$$\begin{array}{l} K_{t}=\frac{C_{UV,0}\left(1-N_{t}\right)-C_{UV,t}}{C_{UV,0}\left(1-N_{t}\right)} \end{array}$$

Time-dependent room-scale inactivation was summarized using a first-order model:


$$\begin{array}{l} 1-K_{t}=\exp\left(-kt\right) \end{array}$$

where *k* is the apparent inactivation rate constant. Characteristic times, including *T*_99_ and *T*_99.9_, were calculated from the fitted *k* values.

For duct-flow single-pass experiments, baseline transport loss across the test section was first obtained from no-UV control trials. Let *C*_*u, c*_ and *C*_*d, c*_ denote the upstream and downstream concentrations in the control trials, respectively. The baseline loss fraction was defined as:


$$\begin{array}{l} N=\frac{C_{u,c}-C_{d,c}}{C_{u,c}} \end{array}$$

Let *C*_*u, UV*_ and *C*_*d, UV*_ denote the upstream and downstream concentrations in the UV-treatment trials, respectively. The UV-attributed single-pass inactivation efficiency was calculated as:


$$\begin{array}{l} E_{w}=\frac{C_{u,UV}\left(1-N\right)-C_{d,UV}}{C_{u,UV}\left(1-N\right)} \end{array}$$

To relate single-pass performance to exposure conditions, a nominal equivalent UV dose per pass was estimated as:


$$\begin{array}{l} D=I_{eq}t \end{array}$$

where *I*_*eq*_ is the nominal equivalent irradiance and t is the nominal residence time within the irradiation region. The equivalent irradiance was estimated by summing the attenuated optical-power contributions of the effective beam segments within one laser curtain:


$$\begin{array}{l} I_{eq}=\frac{P\left(1-R^{m}\right)}{\left(1-R\right)LW} \end{array}$$

where *P* is the effective input optical power for one curtain, *R* is the average reflectivity per reflection, m is the number of effective beam segments, L is the nominal interaction length along the airflow direction, and W is the effective irradiation width. For the duct-flow dose estimation, *P* = 0.77 W, *R* = 0.9884, *m* = 37, *L* = 20 mm, and *W* = 200 mm, corresponding to an effective irradiation area of *LW* = 4,000 mm^2^ and yielding *Ieq*≈5.79 mW/mm^2^.

For three identical curtains arranged in series, the nominal residence time was estimated as:


$$\begin{array}{l} t=\frac{3L}{v} \end{array}$$

where *L* is the nominal interaction length of each curtain along the airflow direction and *v* is the air speed. For example, at *v* = 1.2 m/s, the nominal residence time was 0.05 s, resulting in a nominal estimated dose of 0.2895 mJ/mm^2^, equivalent to 28.95 mJ/cm^2^. Dose values are reported in mJ/mm^2^ and, where tabulated, are also converted to the standard UVGI unit of mJ/cm^2^ using 1 mJ/mm^2^ = 100 mJ/cm^2^.

The LS125 local irradiance measurement was used for on-site verification of the local irradiation level and was not directly used to calculate *Ieq*; accordingly, the nominal LS125 instrument precision was not propagated into the dose estimates. Because a calibrated spatial irradiance map and an independently validated residence-time distribution were not available, the calculated dose values should be interpreted as nominal estimates rather than spatially averaged delivered doses. No formal uncertainty-propagation analysis was performed. Potential sources of uncertainty include spatial non-uniformity of irradiance across the treated cross-section, uncertainties in optical power and mirror reflectivity, variations in optical alignment and attenuation, and heterogeneity in airflow velocity and residence time.

These dose–residence-time relationships are consistent with in-duct UVGI design principles (8, 10). The corresponding descriptive dose-response trend and velocity-dose mapping are provided in Supplementary Figure S2. Because the duct experiment included only four airflow/dose conditions, no fitted duct dose-response kinetic model was used as a main analysis.

### Statistical analysis

Room-scale inactivation data are reported as mean ± standard deviation based on three replicate measurements at each time point. First-order kinetic fitting was performed separately for each curtain configuration using the time-dependent inactivation data and was interpreted as an apparent, descriptive analysis under the tested chamber conditions. Raw replicate data for the room-scale experiments are provided in Supplementary Table S1. Formal confidence intervals for the fitted kinetic parameters were not calculated because of the limited number of observations and the presence of high-inactivation endpoints approaching the upper reporting limit. In addition, no formal inferential comparison of the fitted kinetic parameters was performed between the 96-mm and 200-mm configurations. Accordingly, differences in the fitted rate constants and model-derived characteristic times are interpreted descriptively and should not be regarded as statistically significant differences. Duct-flow single-pass results are reported as measured inactivation efficiencies for each airflow condition. Because the duct experiment included only four airflow/dose conditions, no formal duct dose-response model was fitted.

## Results

### Room-scale aerosol inactivation in a 20 m^3^ enclosure

**Figure 3:**
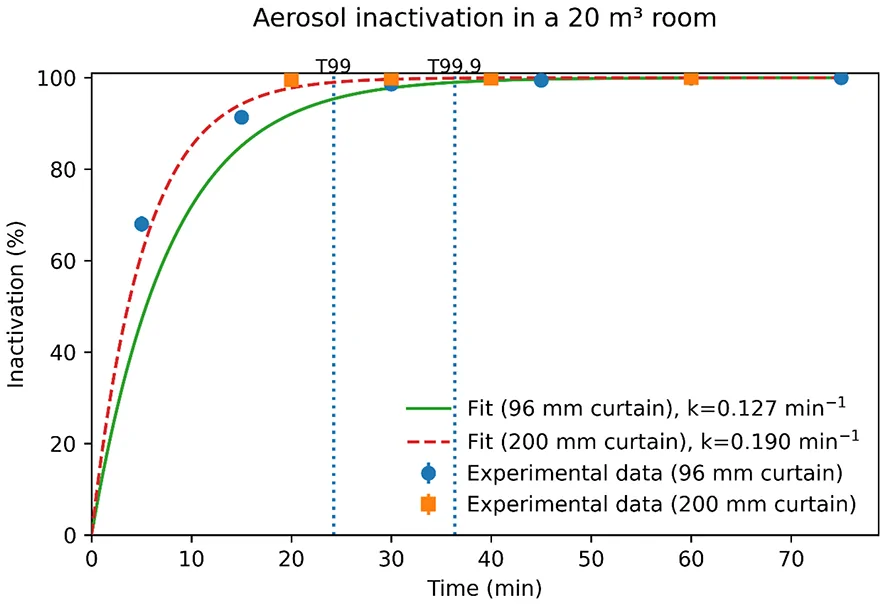
Time-resolved aerosol inactivation in a 20 m^3^ mixed-air enclosure. Markers show experimental inactivation efficiencies for the 96 mm and 200 mm curtain configurations, and curves show first-order fits. Data are presented as mean ± standard deviation from three replicate measurements at each time point.

Time-resolved inactivation of aerosolised Staphylococcus albus was first evaluated under mixed-air conditions in a 20 m^3^ chamber using a single laser-curtain module. For the 96 mm curtain configuration, measurable inactivation was observed within the first few minutes and increased monotonically with operating time (Figure 3). Across replicate measurements, the inactivation level was 68.04 ± 1.74% at 5 min, increased to 91.38 ± 1.15% at 15 min, exceeded 98% by 30 min, and approached ≥99.9% with extended operation. Raw replicate data are provided in Supplementary Table S1.

The 200-mm-wide curtain configuration showed a descriptively faster inactivation pattern than the 96-mm configuration (Figure 3). Inactivation reached 99.55 ± 0.15% by 20 min, 99.93 ± 0.02% by 30 min, and approached ≥99.99% at longer operating times. Descriptively, the 200-mm configuration approached high-level reduction of culturable airborne bacteria more rapidly than the 96-mm configuration.

A first-order inactivation framework provided a concise descriptive summary of the room-scale kinetics for both configurations. The fitted apparent rate constant was numerically higher for the 200-mm-wide curtain than for the 96-mm-wide curtain (0.190 vs. 0.127 min^−1^). Correspondingly, the model-derived characteristic times decreased from T99 approximately 36.3 min and T99.9 approximately 54.4 min for the 96 mm curtain to T99 approximately 24.2 min and T99.9 approximately 36.4 min for the 200 mm curtain, consistent with the observed left-shift in the time-inactivation curves. These parameters should be interpreted as apparent chamber-scale values because the highest inactivation points approached the reporting limit and because independent validation of complete chamber mixing was not performed. No formal statistical comparison was performed between the two fitted rate constants; therefore, the observed difference should be interpreted descriptively.

### Single-pass inactivation in duct flow with stacked laser curtains

Single-pass performance was assessed in a duct-flow testbed using three laser-curtain layers arranged in series, with paired upstream and downstream sampling used to quantify inactivation across the treatment section (Figure 2). Across the tested air speeds, high single-pass inactivation was maintained (Table 2). At 1.2 m/s, the single-pass inactivation efficiency was 99.93 ± 0.02%; at 2.0 m/s, 99.90 ± 0.05%; at 5.0 m/s, 99.38 ± 0.07%; and at 9.6 m/s, 99.00 ± 0.11%. These results indicate that, although increasing air speed reduced the nominal estimated dose per pass, the stacked-curtain configuration preserved ≥99.00% single-pass inactivation across the evaluated flow regime.

**Table 2:** Single-pass inactivation of aerosolised Staphylococcus albus in a duct using a three-layer 266 nm laser curtain. Nominal estimated UV dose is reported in both mJ/mm^2^ and mJ/cm^2^.

Airflow rate (m^3^/h)	Air speed (m/s)	Nominal estimated UV dose (mJ/mm^2^)	Replicate values (%)	Single-pass inactivation, mean ± SD (%)
100	1.2	0.2895	99.94	99.93 ± 0.02
			99.91	
			99.95	
166	2	0.1734	99.84	99.90 ± 0.05
			99.92	
			99.93	
416	5	0.0693	99.31	99.38 ± 0.07
			99.45	
			99.37	
800	9.6	0.0363	99.12	99.00 ± 0.11
			98.96	
			98.92	

To relate duct performance to exposure conditions, the nominal estimated dose per pass was reported for each air speed. The nominal estimated dose decreased from 0.2895 mJ/mm^2^ (28.95 mJ/cm^2^) at 1.2 m/s to 0.0363 mJ/mm^2^ (3.63 mJ/cm^2^) at 9.6 m/s, reflecting the reduced residence time at higher flow speeds. Despite this reduction, the observed single-pass inactivation remained within a narrow high-performance range of 99.00-99.93%. The corresponding descriptive dose-response trend and velocity-dose mapping are provided in Supplementary Figure S2.

### Summary of performance across room and duct scenarios

Taken together, the room-scale and duct-flow experiments demonstrate that the 266 nm multi-reflection laser-curtain concept can be evaluated in two complementary regimes: cumulative treatment in an enclosed mixed-air chamber and single-pass treatment under controlled duct-flow conditions. The room-scale results show that the 200-mm configuration yielded a numerically higher apparent inactivation rate constant and shorter model-derived characteristic times than the 96-mm configuration. The duct-flow results further show that arranging three curtains in series supported ≥99.00% single-pass inactivation over the tested air-speed range, while the dose-based description provides an interpretable link between airflow conditions and nominal estimated exposure.

## Discussion

This study evaluated a compact 266-nm multi-reflection laser-curtain module for airborne bacterial inactivation under two controlled experimental scenarios: cumulative treatment in a mixed-air 20 m^3^ enclosure and single-pass treatment in a duct-flow configuration with stacked curtains. By combining room-scale and duct-flow tests, the study provides process- and device-level validation evidence for a distributed UV laser-curtain architecture. This evaluation strategy is relevant because UVGI performance is known to be sensitive to installation conditions, airflow regimes, irradiance distribution, and maintenance practices (3, 7).

In the room-scale setting, inactivation increased with operating time for both effective curtain widths. The 200-mm configuration yielded a numerically higher fitted first-order rate constant and shorter model-derived characteristic times than the 96-mm configuration. However, confidence intervals for these kinetic parameters were not calculated, and no formal inferential comparison was performed between the two configurations because of the limited dataset and the presence of endpoints approaching the upper reporting limit. Therefore, the differences between the two configurations should be interpreted as descriptive rather than statistically confirmed. In addition, apparent room-scale kinetics should not be interpreted as photoinactivation alone. They also reflect the coupling between airflow mixing, aerosol transport, and the probability that aerosol parcels repeatedly traverse the irradiated region. These factors vary across room geometries, ventilation patterns, and occupancy conditions (1, 8, 9). Therefore, extrapolation from the present 20 m^3^ chamber to real healthcare spaces should be made cautiously and should be supported by further site-specific engineering evaluation.

In the duct-flow setting, the three-layer stacked configuration achieved high single-pass inactivation across the tested air speeds. This result is consistent with in-duct UVGI principles, where UV dose is jointly determined by irradiance, residence time, duct geometry, and airflow velocity (8, 10). Reporting the nominal estimated dose per pass provides an interpretable way to relate airflow conditions to single-pass inactivation, while recognizing that the calculated values do not represent spatially resolved dose measurements. Because only four airflow/dose conditions were tested, the duct-flow results were presented as measured single-pass performance with a descriptive dose-response trend rather than as a fitted kinetic model. This conservative analysis avoids over-parameterisation while still showing that the stacked-curtain configuration maintained high inactivation under the tested flow conditions.

The optical design is a central feature of the proposed module. By folding a 266-nm beam between high-reflectance mirrors, the system converts a single input beam into a distributed irradiation region across the airflow cross-section. The supplementary ray-tracing illustration and optical attenuation measurements provide additional transparency regarding the multi-reflection light path and power loss during repeated reflections. This optical characterization is important because irradiance distribution and residence time are key determinants of UVGI performance in airflow-based systems (8, 10–13). Nevertheless, the present study did not include a calibrated spatial irradiance map across the treated cross-section. The LS125 measurement was obtained only at a local position near the first reflection point and was used for local irradiation verification rather than for determining a spatially averaged irradiance. The reported dose values were therefore calculated from the optical input power, mirror reflectivity, number of effective beam segments, nominal irradiation geometry, and nominal airflow residence time. Consequently, spatial variations in irradiance and residence time could not be incorporated into the dose calculation, and the reported dose values should be interpreted as nominal estimates rather than spatially averaged delivered doses. No formal uncertainty-propagation analysis was performed. Future work should include calibrated irradiance mapping, quantitative uncertainty analysis, and long-term monitoring of optical alignment, mirror reflectivity, and power stability.

Safety and lifecycle considerations are also central to any practical use of 266-nm UVGI. Radiation at this wavelength can injure skin and eyes, and the laser-curtain region must therefore be enclosed, shielded, and controlled by interlocks before operation in occupied or maintenance-accessible environments. The present prototype was tested under controlled laboratory conditions, but systematic UV leakage mapping at access panels, optical joints, air inlet/outlet regions, and worst-case operating conditions was not performed. Future device development should include leakage measurements against applicable occupational exposure limits, fail-safe interlocks, emergency shutoff controls, warning indicators, access restrictions, maintenance procedures with the UV source disabled, and periodic verification of laser output power, mirror reflectivity, optical alignment, and mirror contamination or degradation.

Relative to conventional mercury-lamp or UV-LED UVGI systems, the laser-curtain architecture may offer a compact and geometrically controlled exposure region, but the present study did not perform a direct comparison of dose efficiency, electrical energy per treated airflow, cost, maintenance burden, or lifecycle. Conventional UVGI technologies remain more mature and may be simpler and less costly in many installations, whereas the laser-curtain approach introduces additional optical-alignment, mirror-cleaning, and laser-safety requirements. Therefore, the present data should not be interpreted as evidence of superiority over established UVGI systems; future comparisons should use matched airflow, target organisms, dose metrics, input power, footprint, cost, and maintenance protocols.

Several limitations should be noted. First, the biological evaluation was restricted to aerosolised S. albus, and the nomenclature and surrogate role of this organism limit direct generalization to clinically relevant pathogen classes, including viruses and other bacterial species, whose susceptibility can vary across microbial species, wavelength regimes and exposure conditions (18–21). Second, aerosol particle-size distribution was not measured, and the recovery efficiency of the air-sampling method was not independently validated. Consequently, size-dependent differences in UV susceptibility, aerosol transport and deposition, as well as potential sampling losses, could not be quantified. These limitations may affect the absolute quantitative interpretation of the airborne bacterial concentrations and inactivation efficiencies, although matched no-UV controls were used to reduce the influence of non-UV-related losses (8, 9, 14–16). Future studies should incorporate particle-size-resolved aerosol characterization and sampler recovery validation under the relevant airflow and environmental conditions. Third, the room-scale analysis assumed sufficiently mixed chamber air following fan-assisted circulation; however, complete spatial mixing and the uniformity of airborne bacterial concentration were not independently validated using CFD, tracer measurements, or spatially distributed sampling. Consequently, the fitted room-scale kinetic parameters should be interpreted as apparent chamber-scale values under the tested mixing protocol rather than as intrinsic photoinactivation constants. Fourth, although the duct-flow experiment used a nominal residence-time calculation, CFD or tracer-based validation of airflow distribution and residence-time heterogeneity was not performed; such analyses are important because in-duct UVGI performance depends on duct geometry, air velocity, irradiance distribution and residence time (8, 10, 12, 15, 21). Fifth, the present work did not evaluate long-term operational stability, quantitative UV leakage, maintenance intervals, or direct energy and cost comparisons with conventional mercury-lamp or UV-LED UVGI systems. These aspects are important for translating UV air-disinfection technologies from controlled experiments to practical healthcare environments, where installation conditions, safety management and maintenance practices strongly influence performance (3, 7, 8).

Overall, the findings support further development of compact multi-reflection 266-nm laser-curtain architectures as an experimental air-disinfection approach. The present work demonstrates feasibility in controlled room-scale and duct-flow settings, while future studies should expand biological scope, quantify spatial dose distribution, evaluate airflow-residence-time behavior, verify UV safety controls, and compare operational performance with established UVGI technologies before making healthcare deployment claims (3, 7, 8, 10–17, 21).

## Conclusion

A compact 266-nm multi-reflection laser-curtain module was designed and evaluated for airborne bacterial inactivation in a 20 m^3^ mixed-air chamber and a duct-flow single-pass testbed. Room-scale experiments showed time-dependent inactivation that was described using an apparent first-order framework and showed numerically faster apparent kinetics with the 200-mm configuration than with the 96-mm configuration. Duct-flow experiments using three curtains in series demonstrated high single-pass inactivation across the tested air-speed range. These results support the feasibility of the laser-curtain concept as a controllable UV air-disinfection architecture under laboratory conditions. Further work should expand pathogen testing, quantify spatial irradiance and residence-time distributions, assess UV leakage, long-term optical stability, safety controls, energy use, cost, and maintenance, and compare operational performance with conventional UVGI systems before healthcare deployment claims are made.

## Data Availability

The original contributions presented in the study are included in the article/Supplementary material, further inquiries can be directed to the corresponding author.
