# Supplementary material for: A 266-nm multi-reflection laser curtain for airborne bacterial inactivation in a 20-m3 chamber and a single-pass duct testbed
Source: Front Public Health. 2026 Aug 25;14:1809909. doi: 10.3389/fpubh.2026.1809909 (PMC13550113; doi:10.3389/fpubh.2026.1809909)
Supplement: Supplementary file 1 [file Supplementary_file_1.docx]

**Table S1.** Time-dependent inactivation of aerosolised Staphylococcus albus in a 20 m³ chamber during operation of the UV laser curtain.

| Curtain configuration | Exposure time (min) | Replicate | inactivation (%) | Mean ± SD (%) |
| --- | --- | --- | --- | --- |
| 96 mm curtain | 5 | 1 | 66.07 | 68.04 ± 1.74 |
|  |  | 2 | 69.35 |  |
|  |  | 3 | 68.71 |  |
|  | 15 | 1 | 90.15 | 91.38 ± 1.15 |
|  |  | 2 | 92.43 |  |
|  |  | 3 | 91.55 |  |
|  | 30 | 1 | 98.16 | 98.64 ± 0.44 |
|  |  | 2 | 99.01 |  |
|  |  | 3 | 98.75 |  |
|  | 45 | 1 | 98.85 | 99.47 ± 0.54 |
|  |  | 2 | 99.83 |  |
|  |  | 3 | 99.72 |  |
|  | 60 | 1 | 99.92 | 99.97 ± 0.04 |
|  |  | 2 | 99.99 |  |
|  |  | 3 | 99.99 |  |
|  | 75 | 1 | ≥99.99 | ≥99.99 |
|  |  | 2 | ≥99.99 |  |
|  |  | 3 | ≥99.99 |  |
| 200 mm curtain | 20 | 1 | 99.51 | 99.55 ± 0.15 |
|  |  | 2 | 99.72 |  |
|  |  | 3 | 99.42 |  |
|  | 30 | 1 | 99.94 | 99.93 ± 0.02 |
|  |  | 2 | 99.94 |  |
|  |  | 3 | 99.91 |  |
|  | 40 | 1 | 99.99 | 99.99 ± 0.01 |
|  |  | 2 | 99.99 |  |
|  |  | 3 | 99.98 |  |
|  | 60 | 1 | ≥99.99 | ≥99.99 |
|  |  | 2 | ≥99.99 |  |
|  |  | 3 | ≥99.99 |  |

**Note:** Values reported as ≥99.99% indicate that the measured inactivation reached or exceeded the upper reporting level under the experimental conditions.

**Table S2.** Optical power attenuation during repeated mirror reflections.

| **Number of reflections** | **Measured power (W)** | **Relative power (%)** |
| --- | --- | --- |
| 0 | 0.77 | 100 |
| 2 | 0.76 | 98.7 |
| 4 | 0.75 | 97.4 |
| 6 | 0.73 | 94.81 |
| 8 | 0.71 | 92.21 |
| 10 | 0.68 | 88.31 |
| 12 | 0.66 | 85.71 |
| 14 | 0.64 | 83.12 |
| 16 | 0.62 | 80.52 |
| 18 | 0.61 | 79.22 |
| 20 | 0.58 | 75.32 |
| 22 | 0.57 | 74.03 |
| 24 | 0.54 | 70.13 |
| **Average reflectivity per reflection** | | 98.84 |

**Note:** $P_{0}=0.77$W. Relative power was calculated as $P_{N}/P_{0}\times100\%$. The reflectivity per reflection was estimated as $R_{N}=(P_{N}/P_{0})^{1/N}\times100\%$, and the average reflectivity was calculated from $R_{N}$ values for $N=2$ to $24$.

**Figure S1.** Ray-tracing illustration of the multi-reflection laser-curtain design. (a) Folded optical path generated by repeated reflections between two high-reflectance mirrors. (b) Dense optical coverage formed by the multi-reflection path, creating the laser-curtain irradiation region

**Figure S2.** Supplementary analysis of duct single-pass exposure conditions. (a) Experimental dose-response trend for aerosolised Staphylococcus albus in the duct-flow testbed. The dashed line is included only as a guide to the eye and does not represent a fitted kinetic model. (b) Delivered dose per pass as a function of air speed, calculated from the equivalent irradiance and residence time.
